# Perceptual and quantitative analysis of discoloration of orthodontic elastomeric chains by food

**DOI:** 10.1186/s12903-023-02825-2

**Published:** 2023-02-24

**Authors:** Hyun-Joo Chung, Sun-Ah Lim, Ho-Kyung Lim, Seok-Ki Jung

**Affiliations:** 1 Department of Orthodontics, Korea University Guro Hospital, 148 Gurodong-ro, Guro-gu, Seoul, 08308 Korea; 2 Department of Orthodontics, Graduate School of Clinical Dentistry, Korea University, Seoul, 02841 Korea; 3 Department of Oral and Maxillofacial Surgery, Korea University Guro Hospital, 148 Gurodong-ro, Guro-gu, Seoul, 08308 Korea

**Keywords:** Acceptability threshold, Digital quantitative analysis, Discoloration, Elastomeric chain, Visual analysis

## Abstract

**Background:**

The objectives of this study were to use a digital camera to measure the discoloration of orthodontic elastomeric chains in various immersion solutions over different time periods and to determine the valid acceptability tolerances for color changes in orthodontic elastomeric chains by surveying digital photographs.

**Methods:**

Orthodontic elastomeric chains were applied to the maxillary anterior teeth of nine typodont models. The models were divided into three groups and immersed in the curry, coffee, and wine solutions. The digital images of the elastomeric modules were captured and processed using commercial software after 30 min of immersion, thrice a day, for 28 days. The differences in color changes ($\Delta {\text{E}}$), depending on the type of immersion solution and period, were analyzed using a repeated-measures analysis of variance (ANOVA) test. A web-based survey questionnaire was randomly distributed to 50 respondents for a visual analysis of the elastomeric chain discoloration. The relationship between the surveying score and $\Delta {\text{E}}$ value was analyzed using Spearman’s correlation coefficient. The perceptibility and acceptability of elastomeric chain discoloration ($\Delta {\text{E}}$) based on the type of immersion solutions and periods were analyzed using a regression model.

**Results:**

Significant differences were observed in the discoloration of the elastomeric power chain depending on the immersion solution and period. The amount of discoloration was highest in curry, followed by coffee and wine *(P* < 0.05). The mean discoloration ($\Delta {\text{E}}$) continued to increase over the entire immersion period. There was a significant correlation between visual scoring and discoloration ($\Delta {\text{E}}$) over the entire period, especially in the early stages compared to the later stages (r = 0.918*, P* < 0.05). In 50% of the respondents, the predicted clinically unacceptable discoloration was between 4.46 and 9.98 and in 90% of the respondents, it was between 16.52 and 19.85.

**Conclusions:**

The amount of discoloration was the highest for curry, followed by coffee and wine, and continued to gradually increase during the observation period. Significant differences were found between the color measurements obtained and the visual assessment by observers. The observers varied in their tolerance for perceptibility and acceptability for elastomeric chain discoloration based on the type of dietary media.

## Background

The demand for orthodontic treatment in adults has increased significantly with the support of well-established contemporary orthodontics. These patients have particularly high expectations regarding esthetics, and they wish to maintain an esthetically pleasing appearance of fixed orthodontic appliances during treatment. These concerns have fueled the use of esthetic brackets, wires, and clear elastomeric modules [1]. However, in clinical situations, some of these products are stained by food and drinks, thereby inducing several undesirable changes in their properties, particularly in color. Although most ceramic brackets are resistant to discoloration, elastomeric modules are susceptible to color degradation, which causes a significant esthetic problem [2].

Various methods are currently available for assessing and quantifying these color changes. Although spectrophotometry is considered the gold standard for instrumental color analysis, its application in measuring the color of orthodontic elastomeric modules has some limitations, such as the need for a relatively large measurement area and geometric distortions caused by the curvature of the elastomeric modules. Recent advances in photography and computer science have enabled measurements to be made using simpler and more cost-effective procedures than previous methods [3]. With advancements in technology, it has become possible to acquire images using digital cameras and analyze color data using commercial imaging software. In the case of appropriate calibration protocols and digital cameras, statistically significant correlations were found between the measurements using spectrophotometers and digital cameras for all the International Commission on Illumination (French name: Commission Internationale de 1’Eclarage) (CIE) L*, a*, and b* color coordinates [4].

The practical application of color measurements requires the establishment of parameters that have visual significance; tolerance for perceptibility (magnitude of color difference that is visually detectable); and tolerance for acceptability (magnitude of color difference that is unacceptable to dental esthetics). Opinions of both dentists and patients are important for establishing visual thresholds because of esthetic needs and the long intervals between visits during orthodontic treatment. By identifying the visual thresholds for patients, dentists can benefit from a better understanding of patients’ own observations, leading to improved communication.

Tolerance for visual thresholds can be measured using the color difference at which 50% of the subjects would perceive a color difference (tolerance for perceptibility) or at which 50% of the subjects are willing to redo what was done because of color mismatch (tolerance for acceptability). In many previous studies, tolerance to color differences has been investigated in other dental materials such as composite resin disks, porcelain disks, and metal-ceramic crowns [5–10]. However, although previous studies had information on color change, few studies investigated how the change was perceived by patients and whether it was acceptable.

Therefore, this study aimed to measure the color changes in orthodontic elastomeric modules after exposure to three types of high-pigment foods and beverages (curry, coffee, and wine) in vitro over 4 weeks and to correlate the measurements with actual visual assessments. Through this, this study also aimed to evaluate a visual threshold for perceptibility and acceptability of color changes of the elastomeric modules.

## Methods

### Sample preparation

Nine typodont models were prepared, and ceramic brackets (Crystalline 7 ceramic bracket; Tomy International Inc., Tokyo, Japan) were bonded to six maxillary anterior teeth. Using 0.010″ stainless-steel ligatures, a 0.017″ × 0.025″ stainless-steel archwire (Ormco, Glendora, CA, USA) was ligated to the brackets. Elastomeric modules (Ormco Generation II Power Chain; Ormco, Glendora, CA, USA) were placed over the brackets. The elastomeric chain was a transparent product made of 4,4′-methylenediphenyl diisocyanate.

The nine typodont models were divided into three experimental groups: coffee (Maxim Mocha Gold Mild Coffee Mix, Dong Suh Co., Ltd, Seoul, Korea), red wine (Cabernet Sauvignon, Viu Manent Winery, Santiago de Chile, Chile), and curry (Three Minutes Instant Curry, Ottogi, Seoul, Korea). All products were finished products in liquid form. Also, all solutions were prepared according to the manufacturer’s instructions. To simulate oral conditions, all models were immersed in the prepared solution following the designated experimental groups thrice a day. After 30 min of immersion, to mimic the oral environment with saliva, samples were washed with distilled water. After that, in order to prevent cross-discoloration due to the water on the surface, samples were lightly dried with a paper towel. The samples were stored at 37 °C in artificial saliva (Taliva, Hanlim Pharm, Seoul, Korea) between immersion sessions. Digital images of the samples were taken on days 1, 3, 5, 7, 9, 11, 13, 15, 21, and 28.

### Digital image acquisition and analysis

A commercial digital single-lens reflex camera (Sony NEX-6, SONY Corp., Tokyo, Japan) was used to acquire digital images of the samples. The camera was set in manual mode, allowing a shutter speed of 1/5 s with an aperture of F32. The International Organization for Standardization (ISO) speed was set to 200 with 1:1 lens magnification [4]. The camera was fixed on a copy stand to provide an optical setup with 0° observation of the object. Digital images were taken in a dark room with two fluorescent tubes (Tornado 20 W CDL E27, Philips Electronics Korea Ltd., Seoul, Korea) as the only light sources. The fluorescent tubes were fixed at an angle of 45°, facing each other at a distance of 45 cm from the platform.

After digital image acquisition, color analysis was performed using commercial imaging software (Adobe Photoshop, version 7.0, Adobe Systems Inc., San Jose, CA, USA). Digital images were loaded into the software, and two areas per tooth (an average 5 × 5 pixels) were randomly selected. The CIE L*, a*, and b* values of each area were obtained (L = degree of lightness; a, b = positions on the red or green and yellow or blue axes (+ a = red, − a = green; + b = yellow, − b = blue). The color difference ($\Delta {\text{E}}_{{{\text{ab}}}}^{*}$) was calculated as $\Delta {\text{E}}_{{{\text{ab}}}}^{*}$ = $\left[ {\left( {\Delta {\text{L}}^{*} } \right)^{2} + \left( {\Delta {\text{a}}^{*} } \right)^{2} + \left( {\Delta {\text{b}}^{*} } \right)^{2} } \right]^{1/2}$.

### Visual assessments

An online survey tool (Qualtrics^XM^) was used to investigate the visual thresholds for perceptibility and acceptability. The survey questionnaire comprised 33 original photographs and 6 duplicates each taken on days 0, 1, 3, 5, 7, 9, 11, 13, 15, 21, and 28, which were randomly arranged. Authors wanted to observe the tolerance of elastic discoloration in the esthetic perspective of the general public, the subject of observer was set to the general public. For the observer criteria, the age of the observer was limited to 20–40 years old, and non-dental workers without color blindness or color weakness were targeted. A set of questionnaires with digital images of the elastomeric modules over 28 days were shared with observers. The observers were asked to score the discoloration of the elastic modules (0 = no discoloration; 1 = mild discoloration; 2 = moderate discoloration; and 3 = severe discoloration) in every image and determine whether the elastomeric modules needed replacement. The visual threshold for perceptibility was calculated at 50% and 90% perceptibility based on a score of 2. Based on the need for replacement, the visual threshold for acceptability was calculated at 50% and 90%.

### Statistical analysis

All statistical analyses were performed using IBM SPSS Statistics for Windows, version 25 (IBM Corp., Armonk, N.Y., USA). The differences in color change according to the type of immersion solution and immersion period were analyzed using a repeated-measures analysis of variance. Normality and equal variance were observed for the measured data. Tukey’s test was performed as a post hoc test. Spearman’s correlation test was used to analyze the correlation between the measured color differences and the visual assessments. Additionally, regression analysis was performed to determine the visual threshold values for perceptibility and acceptability. Two randomly chosen images were duplicated and provided to the participants to test their reliability. Individual reliability was calculated using the Cohen’s kappa test. All statistical analyses were performed at a 95% confidence level.

## Results

**Fig. 1:**
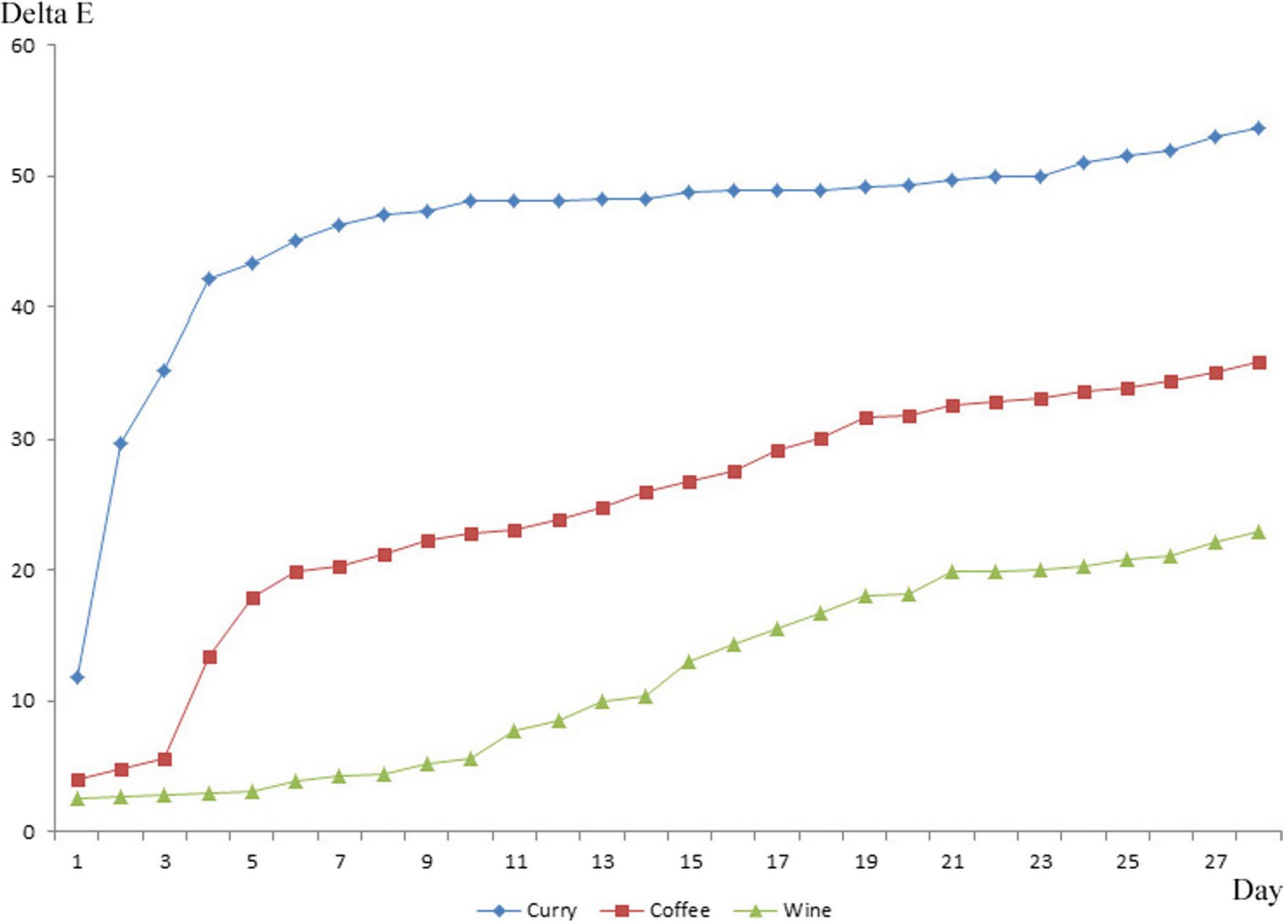
Discoloration of elastomeric chains after immersion in the curry, coffee, and wine solutions

The measured color changes ($\Delta {\text{E}}_{ab}^{*}$) according to the immersion period and immersion solution are shown in Fig. 1. The color changes tended to increase during the immersion period (*P* < 0.01) (Table 1, Fig. 1). The color changes showed a statistically significant variance between the immersion solutions as follows: curry > coffee > wine (*P* < *0.01*), and these differences were maintained at all times (*P* < *0.01*) (Tables 1, 2). Notable color changes were observed in the curry, coffee, and wine solutions on days 1–3, 3–28, and 14–21 (*P* < 0.05) (Table 1).

**Table 1:** Differences between intergroup and intragroup in terms of discoloration ($\Delta {\text{E}}$ value) by immersion period

Time (day)	Curry		Coffee		Wine		F
	Mean ± SD	t	Mean ± SD	t	Mean ± SD	t	
1	11.82 ± 1.17		4.00 ± 1.99		2.62 ± 0.49		39.729*
3	35.32 ± 5.92	− 5.769**	5.64 ± 0.60	− 2.012	2.77 ± 1.23	− 0.176	79.089*
7	46.25 ± 4.00	− 3.442	20.27 ± 1.93	− 16.856*	4.25 ± 0.94	− 2.870	195.709*
14	47.21 ± 5.61	− 0.810	26.02 ± 0.96	− 6.018**	10.36 ± 2.01	− 3.761	84.425*
21	49.72 ± 2.22	− 0.977	32.64 ± 1.06	− 10.410*	19.85 ± 2.21	− 8.741**	184.749*
28	53.74 ± 1.77	− 3.225	35.98 ± 0.42	− 9.052**	22.93 ± 0.32	− 2.113	629.352*

*SD* standard deviation

*P < 0.01; **P < 0.05

**Table 2:** Repeated-measures ANOVA for elastomeric chains discoloration ($\Delta {\text{E}}$ value) within factors and between factors

	Source	Type III sum of square	*df*	Mean square	F	*p*-value
Within factors	Time	4376.360	4	1094.090	206.364*	0.000
	Food*Time	1117.680	8	139.710	26.352*	0.000
	Error	127.242	24	5.302		
Between factors	Intercept	4063.519	1	4063.519	1297.510*	0.000
	Between factors	1414.633	2	707.317	225.851*	0.000
	Error	18.791	6	3.132		

*ANOVA* analysis of variance, *df* degrees of freedom

*P < 0.01

Statistical significance was found in the measured color differences and visual assessment scores (r = 0.918, *P* < 0.01) (Table 3).

**Table 3:** Relationship between surveying score and $\Delta {\text{E}}$ value

	Score	$\Delta {\text{E}}$ value
*Spearman’s rho*		
Score	1.000	
$\Delta {\text{E}}$ value	0.918*	1.000

*P < 0.01

**Fig. 2:**
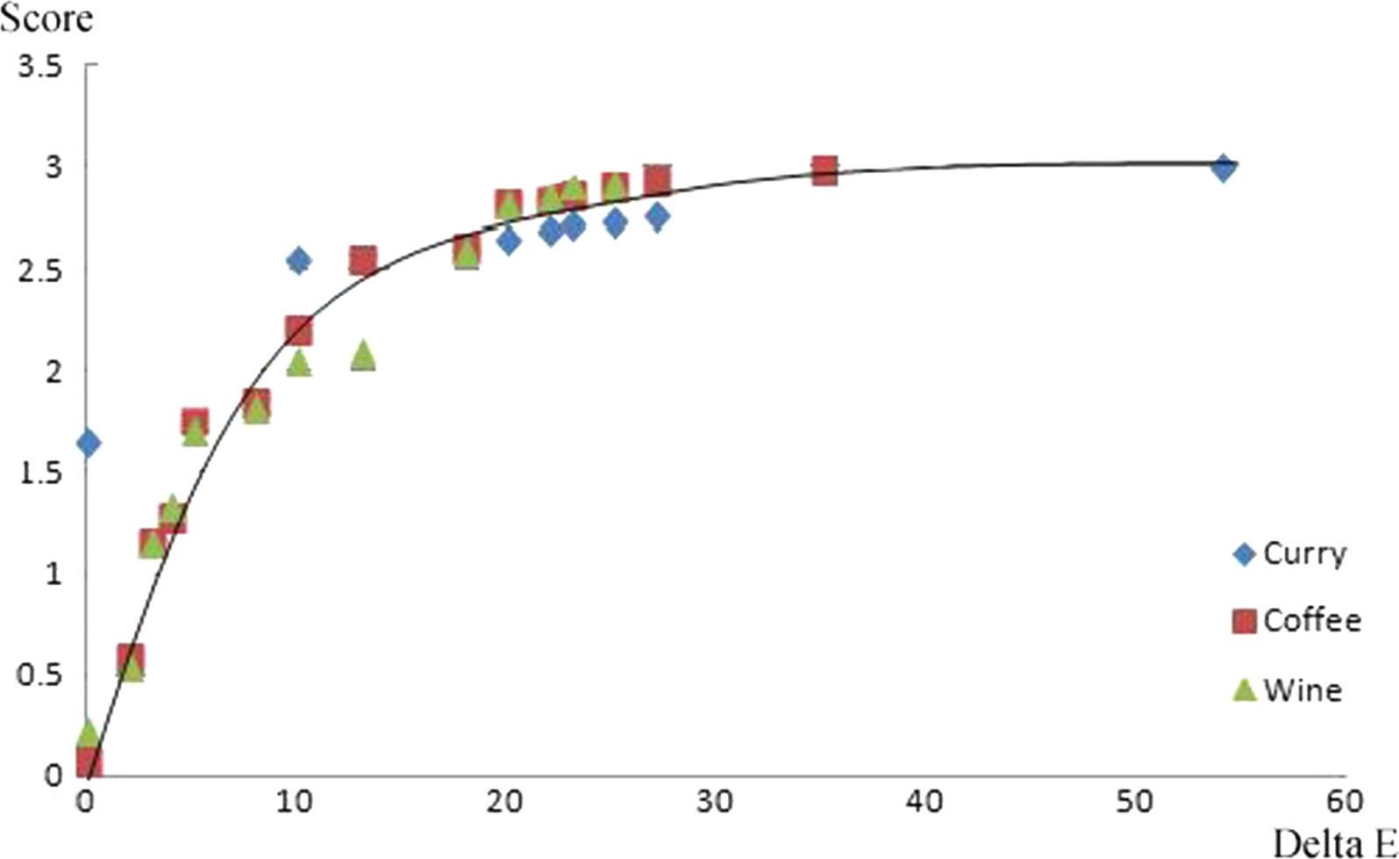
Graph showing the relationship between surveying score and $\Delta {\text{E}}$ value

**Fig. 3:**
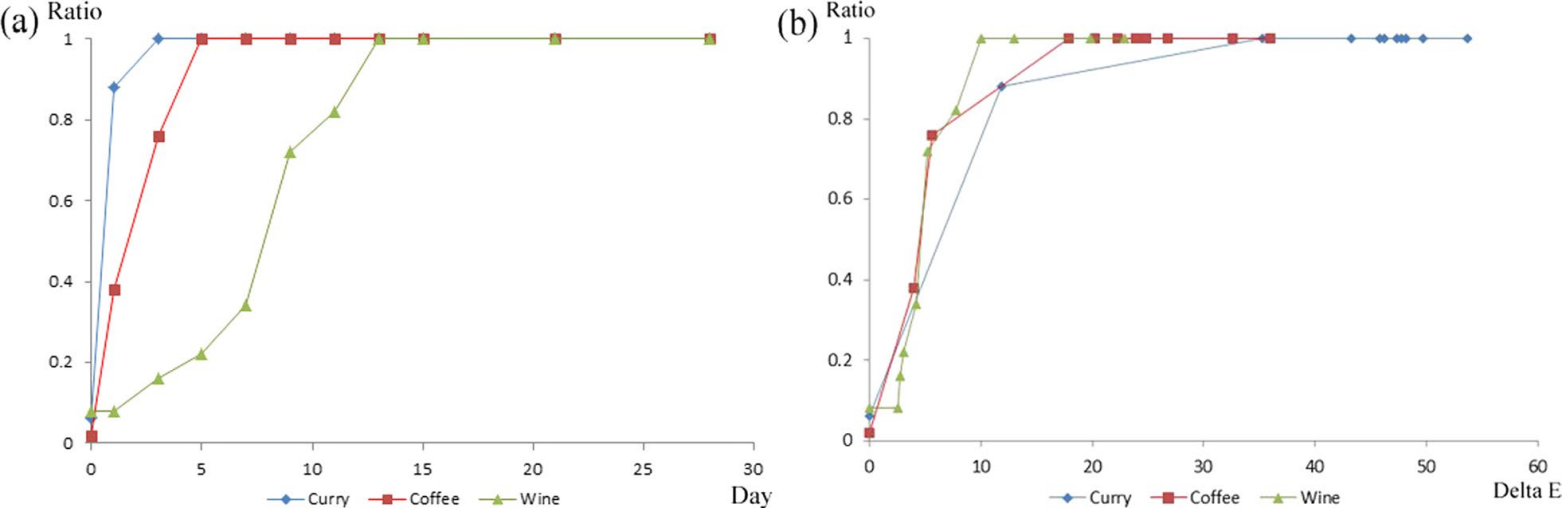
**a** Perceptibility ratio as a function of immersion period in the curry, coffee, and wine solutions, **b** Perceptibility ratio as a function of acceptability elastomeric chains discoloration ($\Delta {\text{E}}$ value) in the curry, coffee and wine solutions

Although the overall scores tended to increase during the early stage of discoloration, with a high $\Delta {\text{E}}$, they showed relatively no change during the latter stage of discoloration, with a low $\Delta {\text{E}}$ (Fig. 2). When scoring data was plotted as a function of color change ($\Delta {\text{E}}$) and immersion period, the color differences for 50% perceptibility in the curry, coffee, and wine solutions were calculated as 6.34, 4.52, and 4.65, respectively. These values correspond to the immersion periods of 0.54, 1.63, and 7.84 days in the curry, coffee, and wine solutions, respectively. The color differences for 90% perceptibility in curry, coffee, and wine solutions were 15.74, 12.79, and 8.76, respectively. These values correspond to the immersion periods of 1.33, 4.17, and 11.89 days in the curry, coffee, and wine solutions, respectively (Fig. 3, Table 4). Perceptibility of discoloration ($\Delta {\text{E}}$) is the result of the perceptibility according to $\Delta {\text{E}}$, and perceptibility of day is the starting date when discoloration is recognized.

**Table 4:** Acceptable discoloration changes and immersion period in respondents

	Ratio (50%)	Ratio (90%)
*Perceptibility of discoloration* ($\Delta {\text{E}}$)		
Curry	6.34	15.74
Coffee	4.52	12.79
Wine	4.65	8.76
*Perceptibility of day*		
Curry	0.54	1.33
Coffee	1.63	4.17
Wine	7.84	11.89
*Acceptability of discoloration* ($\Delta {\text{E}}$)		
Curry	6.34	16.52
Coffee	4.46	17.90
Wine	9.98	19.85
*Acceptability of day*		
Curry	0.54	1.40
Coffee	1.56	5.00
Wine	13.00	21.00

**Fig. 4:**
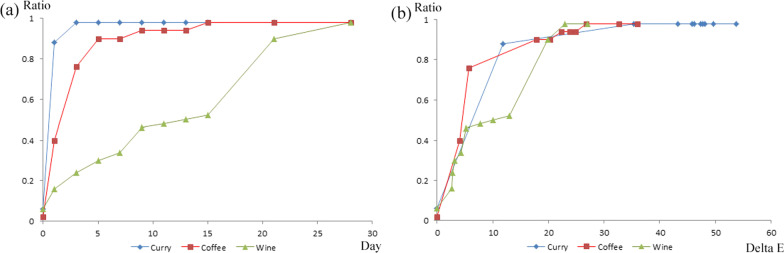
**a** Acceptability ratio as function of immersion period in the curry, coffee, and wine solutions, **b** Acceptability ratio as function of acceptability elastomeric chains discoloration ($\Delta {\text{E}}$ value) in the curry, coffee, and wine solutions

The threshold for acceptability was assessed by plotting the ratio of observers with the need for replacement with the color change ($\Delta {\text{E}}$) and immersion period. The color differences for 50% acceptability in the curry, coffee, and wine solutions were 6.34, 4.46, and 9.98, respectively. The corresponding immersion periods in the curry, coffee, and wine solutions were 0.54, 1.56, and 13 days, respectively. The color differences for 90% acceptability in curry, coffee, and wine solutions were 16.52, 17.90, and 19.85, respectively. The corresponding immersion periods in the curry, coffee, and wine solutions were 1.4, 5, and 21 days, respectively (Fig. 4, Table 4). When comparing the tolerances for perceptibility and acceptability, perceptibility tolerance was smaller than or similar to acceptability tolerance, especially at the 90% ratio. Upon closer examination, the tolerance for perceptibility and acceptability were found to be similar in the curry solution. The tolerance for acceptability was found to be smaller than that for perceptibility in the coffee solution; in contrast, tolerance for perceptibility was smaller than that for acceptability in the wine solution. Further, the observers showed statistically significant intra-rater reliability (Table 5).

**Table 5:** Agreement within 50 people on the four photographs representing acceptability $\Delta {\text{E}}$ value

Variables	Kappa	Agreement (%)	*P*-value
Curry	0.540	82.0	*P* < 0.001
Coffee	0.499	94.0	*P* < 0.001
Wine	0.634	94.0	*P* < 0.001

## Discussion

In general, elastomeric orthodontic modules are composed of polyurethane, which is produced by the condensation polymerization of isocyanates and polyols. Polyurethane surface characteristics reported in the literature are as follows. The water contact angle was about 73.1 ± 0.5, the total surface energy was 47.86 ± 0.09 mN/m, the disperse adhesion was 53.1, and the polar adhesion was 47.1 [11]. Polyurethanes are not inert; hence, they are prone to chemical degradation through hydrolysis with heat, moisture, and enzymatic reactions. Accelerated aging followed by chemical degradation of polyurethane has been reported in ethanol–water solutions [12]. Therefore, a wine solution with an alcohol content of 13% was expected to cause more chemical aging than curry or coffee solutions. Although the wine solution had the highest alcohol content, our study found more discoloration in the curry and coffee solutions. This implies that mechanical staining is more influential than chemical ageing. In addition, previous studies have mentioned the occurrence of chemical degradation during the first few hours as the primary source of discoloration, followed by mechanical staining during the later stages [4]. Therefore, even in the wine solution, mechanical staining was a more dominant factor than chemical staining because a notable color change in the wine solution was reported from days 14–21 toward the end of the immersion period. Hence, the color changes of elastomeric modules can be estimated using the mechanical staining ability and number of exogenous pigments present in foods and drinks rather than their chemical composition.

This study was performed using stretched elastomeric modules to simulate clinical conditions. Although stretching itself, to some degree, can result in the degradation of orthodontic elastomeric modules, ethanol–water solutions are known to accelerate aging and degradation more than stretching [12]. This implies a higher dependence of the degradation on the environment than on the extension state of the module.

The most valuable aspect of this study was the attempt to relate numerical measurement values to patient-related outcomes [13]. The predicted values for the observers’ tolerance for 50% perceptibility and acceptability for the curry, coffee, and wine solutions were 6.34 $\Delta {\text{E}}$ (0.54 days) and 6.34 $\Delta {\text{E}}$ (0.54 days), 4.52 $\Delta {\text{E}}$ (1.63 days) and 4.46 $\Delta {\text{E}}$ (1.56 days), and 4.65 $\Delta {\text{E}}$ (7.84 days) and 9.98 $\Delta {\text{E}}$ (13 days), respectively. These values were greater than those reported in the previous studies, with the tolerance for perceptibility ranging from 1.2 to 3.7 $\Delta {\text{E}}$ and the tolerance for acceptability ranging from 2.7 to 6.8 $\Delta {\text{E}}$ [9, 10, 14–19]. These differences might be because of the methodological variance or the lack of clinical experience or additional training in the randomly chosen observer groups. For the curry solution, the observers indicated replacement within a relatively short time after perceiving the discoloration. The observers’ psychological discomfort due to discoloration from the curry solution is presumed to be high, considering the powerful staining ability of the curry solution (sudden rise in $\Delta {\text{E}}$ during the early stage and high $\Delta {\text{E}}$ throughout the immersion period). Although the observers perceived color changes at similar thresholds for coffee and wine solutions, they indicated earlier replacement for the coffee solution. This accounts for the observers’ varied psychological discomfort with different types of solutions and the distinctive late discoloration behavior of wine solutions. Moreover, coffee solutions are usually consumed at high temperatures, which cause additional degradation of the elastomeric modules during each exposure.

As discoloration is an inevitable event, clinicians should share the concerns of patients and try to reduce their discomfort [20]. Several attempts have been made to increase the resistance to discoloration by changing the microstructure of elastomeric modules. Although increasing the density of elastomer reticulation reduces porosity and increases the resistance to discoloration, it also increases stiffness, which is not ideal for orthodontic use [12]. Existing elastomeric modules from multiple companies are known to have slightly different chemical compositions and mechanical properties with varied abilities to resist discoloration in different categories of dietary media [21]. If changing elastomers is believed to bring about a clinically meaningful aesthetic difference, various elastomers can be tried according to the patient’s dietary habits, despite the clinician’s preference and inefficient stock management. In addition, a NiTi coil spring can be used as a substitute for elastomeric modules. However, it may aggravate aesthetic problems owing to its metallic color and give rise to oral hygiene problems. Therefore, if the patient cannot tolerate aesthetic issues, lingual orthodontic appliances or clear aligner treatment from the treatment planning stage is highly recommended. The visual threshold values for perceptibility and acceptability described in this study can be used as a reference to examine the esthetic demands of patients in advance. Additionally, instructions regarding the influence of dietary media on discoloration should be emphasized.

Although the present study was carried out under carefully designed in vitro conditions, some limitations in simulating the complex intraoral environment, including saliva, metamerism, background (by tooth, bracket, and archwire), and oral hygiene of the patient could remain [22–24]. Moreover, because discoloration can be affected by the immersion period, frequency, and total duration of exposure to foods and drinks, the design of this experiment did not fully realize the clinical environment. In addition, this study was carried out by tracking the color change of the elastomeric module. The state of the elastomeric chain, in which nothing has been done previously, can be regarded as a control. However, it would have been better if a control group such as water was used in the experimental data. Even though there is a significant correlation between measurements of color using a spectrophotometer and digital camera, the aforementioned limitations could cause some differences in its actual clinical application. Despite these limitations, many clinical implications for the discoloration pattern of orthodontic elastomeric modules could be identified through this study.

## Conclusions

The discoloration of the elastomeric modules varied according to the immersion solution (curry > coffee > wine) and period (increased with time). It is presumed that this discoloration was mainly caused by mechanical staining rather than chemical aging. Statistical significance was found between the color measurements obtained and the visual assessments made by the observers. The observers showed varied tolerances in the perceptibility and acceptability of discoloration based on the type of dietary media. Visual threshold values for perceptibility and acceptability can be used as a reference to examine the esthetic demands of patients in advance.

## Data Availability

The datasets used and analyzed during the current study are available from the corresponding author upon reasonable request.
